# Contrast enhancement of biological nanoporous materials with zinc oxide infiltration for electron and X-ray nanoscale microscopy

**DOI:** 10.1038/s41598-017-05690-6

**Published:** 2017-07-19

**Authors:** L. E. Ocola, V. Sampathkumar, N. Kasthuri, R. P. Winarski

**Affiliations:** 1 Argonne National Laboratory, Center for Nanoscale Materials, Argonne, 60543 USA; 2 University of Chicago, Department of Neurobiology, Chicago, 60637 USA; 3 Argonne National Laboratory, Nanoscience and Technology Division, Argonne, 60543 USA

## Abstract

We show that using infiltration of ZnO metal oxide can be useful for high resolution imaging of biological samples in electron and X-ray microscopy. The method is compatible with standard fixation techniques that leave the sample dry, such as finishing with super critical CO_2_ drying, or simple vacuum drying up to 95 °C. We demonstrate this technique can be applied on tooth and brain tissue samples. We also show that high resolution X-ray tomography can be performed on biological systems using Zn K edge (*1s*) absorption to enhance internal structures, and obtained the first nanoscale 10 KeV X-ray absorption images of the interior regions of a tooth.

## Introduction

Most biological materials have problems with charging when imaging with electrons and low contrast when imaging with hard X-rays. Standard protocols for sample preparation include coating techniques, using sputtering of a conductive metal such as gold, platinum, or gold/palladium alloy, or non-coating perfusion techniques using glutaraldehyde, tannic acid and osmium tetroxide^1^. Another method to image biological samples with minimum sample preparation is via environmental electron scanning microscopy, which requires the use of specially designed electron microscopes that use differential pumping to allow for charge dissipation at the sample surface. In this paper we will focus solely on sample preparation protocols for use in standard scanning electron microscopes and x-ray microscopes.

Sputter coating a metallic thin film of 20 nm, or more, allows for a good grounding path for charge dissipation, and is relatively faster than the non-coating method. However, because the technique is a direct line-of-sight deposition, the film will not deposit conformally and may distort any nanoscale features on the surface. Finally, sputter coating is only a surface preparation technique, i.e. any further sub-surface analysis of the sample is no longer possible. In spite of this limitation papers concerned with imaging with electron microscopy still use metal coating to cover the surface of interest^2, 3^.

The perfusion method allows for cell fixation and is conductive enough to not need a metal thin film coating if a tissue sample is thin enough for the electrons to deposit most of their energy in a conductive substrate under the sample. The tissue is first fixed with glutaraldehyde, then treated. The main fixation methods use a series of solutions of osmium tetroxide (OsO_4_), and thiocarbohydrazide, or solutions of  tannic acid, Arginine, and OsO_4_^4^. Perfusion of OsO_4_ increases the electrical conductivity in cells^5^ and it has been part of standard sample preparation techniques for high resolution electron microscopy studies (>30 KX magnification) of biological samples since the early days of the field^5, 6^. However, the method is slow, laborious, and has limitations on how far the solutions will diffuse into the sample, how much OsO_4_ adheres to the tissue, and the possibility of cell distortion due to swelling^7^.

In this paper we describe an alternative method that can be used for both charge dissipation in electron microscopy and as a contrast enhancer for X-ray microscopy. The method uses a technique similar to atomic layer deposition (ALD), but with different conditions. This variant of ALD has been reported in the literature under different names such as: multi-pulse infiltration (MPI)^8^, sequential infiltration synthesis (SiS)^9^, and sequential vapor infiltration (SVI)^10^. In this paper we will refer to the ALD infiltration method as SiS. The SiS method utilizes similar concepts of ALD, but with a significant difference in process exposure times, pressure, and purpose. The purpose is to allow precursor gases infiltrate a porous material so that they can react inside of the material. Another characteristic of SiS is that it operates at low temperatures, below the glass transition temperature of polymers^11^. The first material synthesized on polymers with low temperature ALD was Al_2_O_3_^11^. Conductive metal oxides that can be synthesized at temperatures amenable with biological samples are ZnO^12–14^, and SnO^15^. The key aspects for biological samples to be amenable to the SiS process is that they be fixed, dry, porous, and vacuum compatible.

## Results

We will describe the use of SiS of ZnO as a means to address both charge dissipation in electron microscopy and contrast enhancement for X-ray microscopy. With SiS ZnO infiltration^16^ it is feasible to coat a 2–3 nm thick conductive metal oxide film throughout a porous biological sample, and image both the surface *and the interior of a* sample *without any further sample recoating*. ZnO is a conductive oxide and the gaseous precursors required for its synthesis (water vapor and diethylzinc) easily penetrate nanoporous materials that exhibit water intake^16^. The infiltration depth mainly will depend on the porosity of the material, the residence time the gaseous precursors have to diffuse through the porosity, and the affinity of the precursor molecules to the material surface. Examples of infiltration depth experiments are shown in Figs S1 and S2 in the Supplementary Information section.

The samples imaged by electron microscopy were a canine tooth from a dog, and brain tissue that was fixated with osmium and supercritically dried ahead of time. Both samples were pre-treated in a vacuum oven, initially at room temperature, ramped to 95 °C, and then baked at 95 °C for several hours. The temperature of the vacuum pre-treatment matches that of the SiS ZnO process used in our Arradiance Gemstar ALD tool. The actual SiS process conditions have been extensively described elsewhere^16^. Both tooth and brain tissue samples were inspected by optical microscopy before and after the vacuum oven pre-treatment to determine if the samples were compatible with the SiS process. Given that no sample changes were observable, we proceeded with running a SiS ZnO process of 18 cycles of [H_2_O:DEZ], which would be the equivalent of a 2–3 nm coating on a flat surface. No further processing was needed for both electron and X-ray microscopy imaging. Control experiments for the tooth and brain tissue samples are reported in the Supplementary Information section. This includes EDS data, and SEM image comparison between untreated and SiS ZnO treated samples, highlighting the advantage of SiS ZnO as an SEM imaging charge dissipation material.

### Electron imaging of a SiS ZnO treated tooth sample

**Figure 1:**
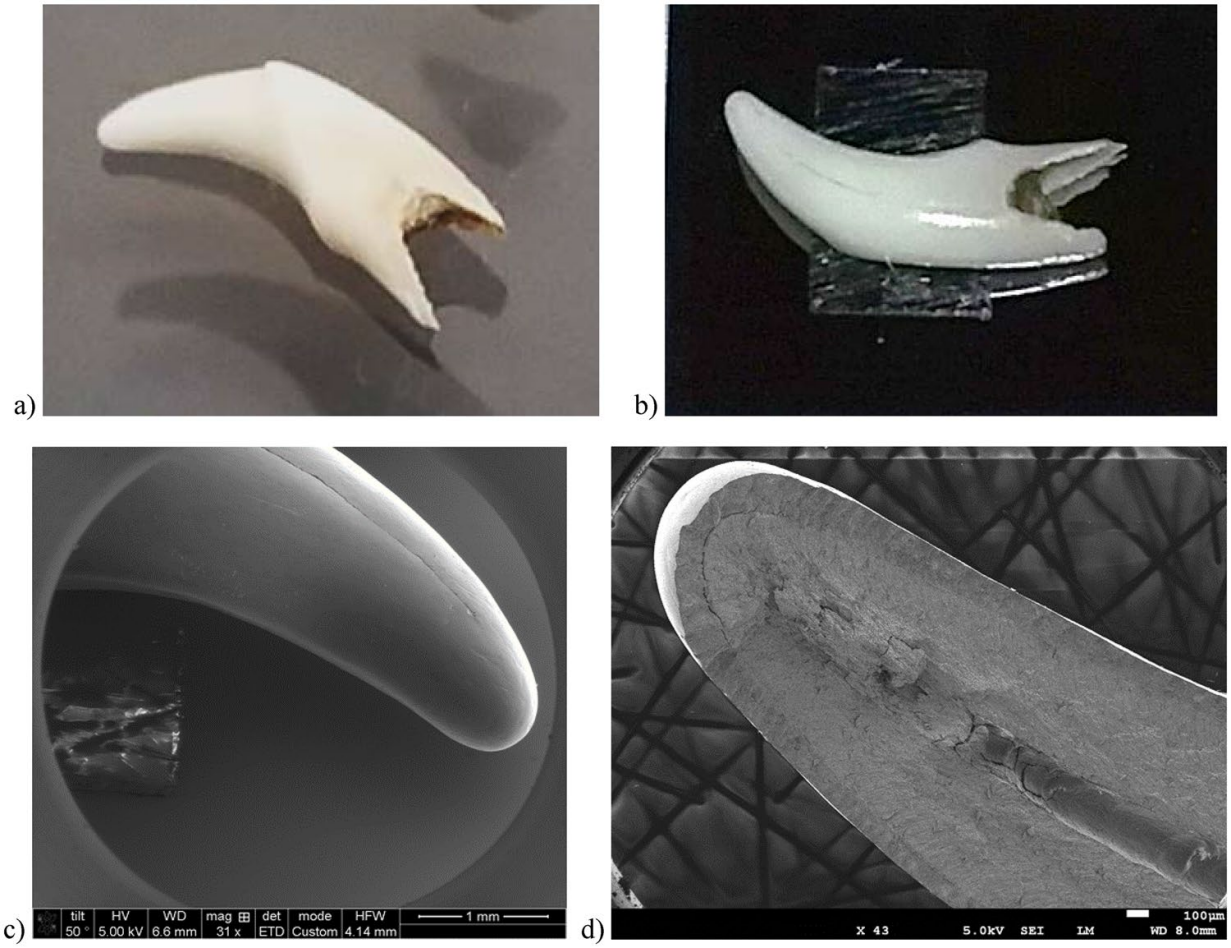
Optical and scanning electron micrographs of SiS ZnO treated tooth sample. (**a**) Photo of tooth sample before fixing on silicon piece carrier. (**b**) Photo of tooth sample after SiS ZnO treatment, showing double sided carbon tape underneath. (**c**) Tooth tip imaged prior to breaking open the tooth along observable crack. (**d**) Same tip after breaking open tooth along crack region.

The sequence for imaging a tooth sample is as follows. First, the tooth was fixed in place with double-sided carbon tape onto a piece of silicon wafer for handling, Fig. 1b. After 18 cycles of SiS ZnO, the sample was placed whole into a field emission scanning electron microscope (FEI Dual Beam FESEM, JEOL 7500 FESEM). Initial observation of the tooth in the electron microscope showed a prominent crack that traveled the length of the tooth, Fig. 1c. Given that it would not be highly remarkable to report on images of the surface of the tooth, we decided to break the tooth along the crack and image inside the tooth, where only diffused ZnO precursors would be able to reach, Fig. 1d. As can be observed, we clearly see the interfaces of the enamel, dentin, and pulp regions with clarity.

**Figure 2:**
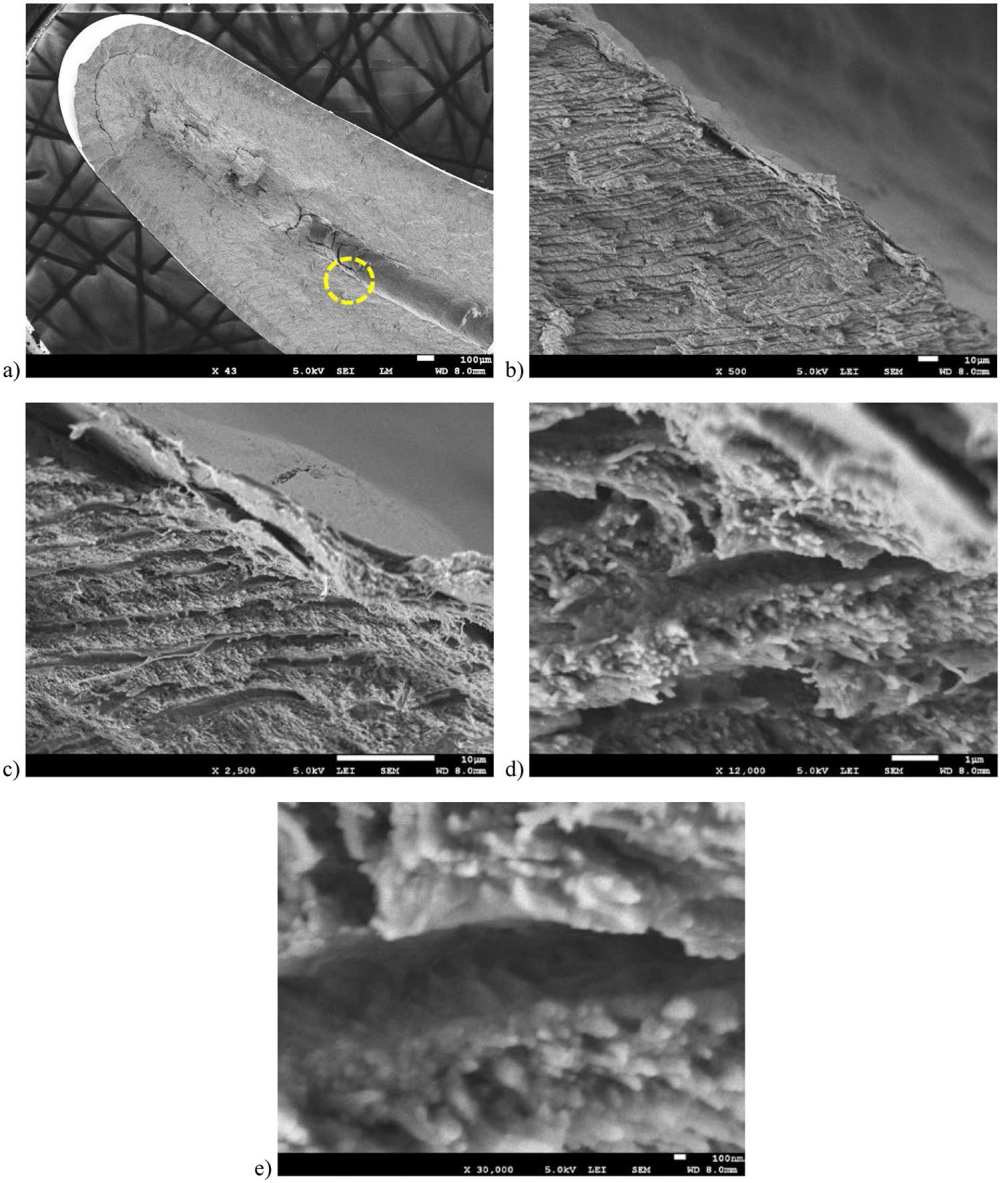
SEM micrographs of a SiS ZnO treated tooth sample with increasing magnification. (**a**) Image taken at 43 X magnification with dashed circle highlighting region of interest. Scale bar is 100 μm. Images (**b**–**e**) are all centered on the same spot. (**b**) SEM micrograph at 500 X magnification. Scale bar is 10 μm. (**c**) SEM micrograph at 2.5 KX magnification. Scale bar is 10 μm. (**d**) SEM micrograph at 12 KX magnification. Scale bar is 1 μm. (**e**) SEM micrograph at 30 KX magnification. Scale bar is 100 nm.

Further inspection with higher resolution images are shown in Fig. 2. The magnifications range from 43 X (Fig. 2a) to 30 KX (Fig. 2e). Thus, we prove that we can observe sub-100 nm dentin tubules and smaller structures that are over 600 microns from the tooth surface without the need of a second metal thin film coating or a complicated perfusion fixation process. Images in Fig. 2 highlight the structure inside the tubules that had been broken in half during sample process.

**Figure 3:**
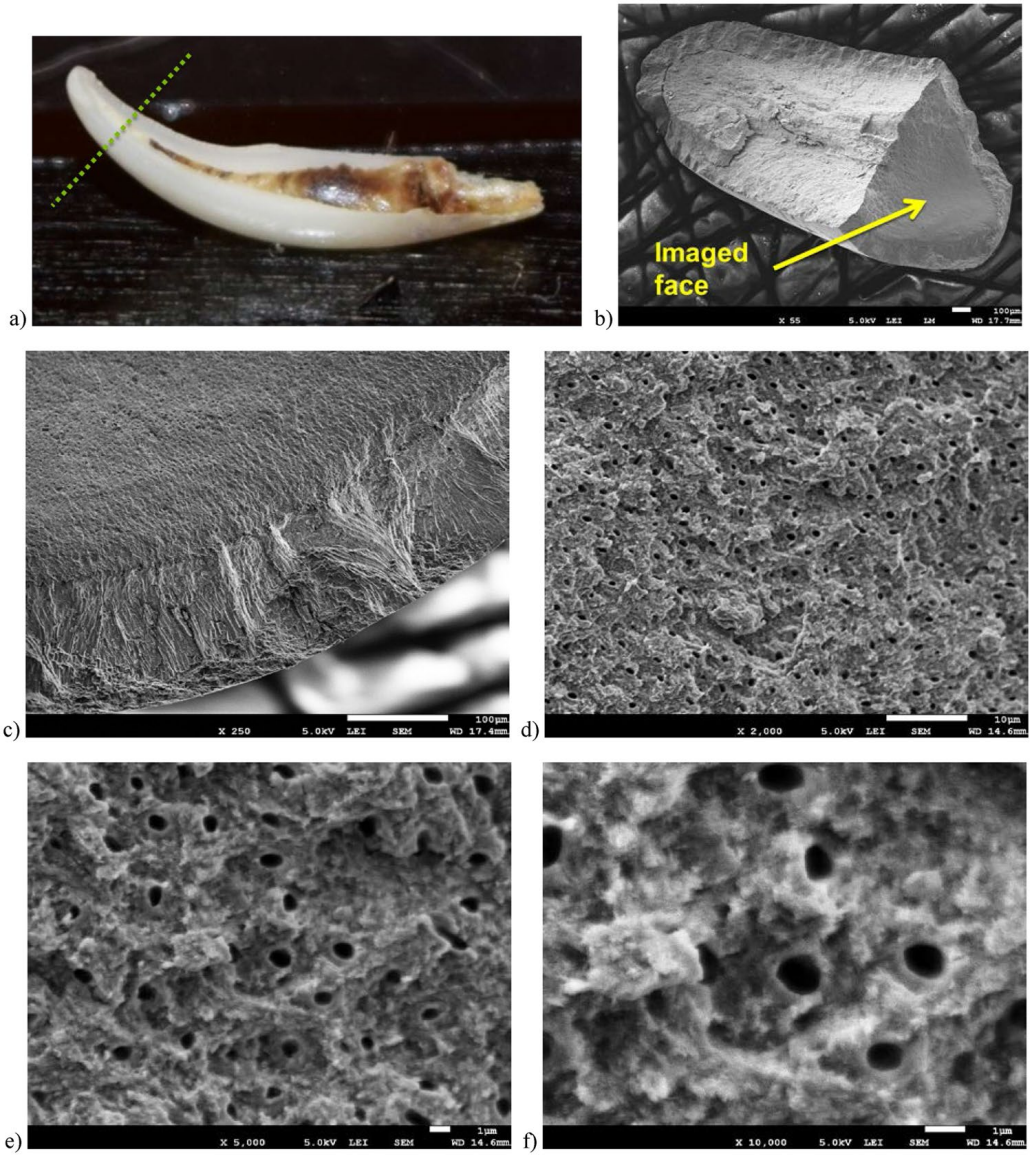
Images of tooth sample after cracking open the first time and then after breaking tip and SEM micrographs of the interior surface of the tooth sample. (**a**) Optical image indicating where tip was broken. (**b**) SEM micrograph of broken tip and indication of imaged face for images (**c**–**f**). (**c**) Image of enamel – dentin interface at 250 X magnification. Scale bar is 100 μm. (**d**) Image of denting region at 2 KX magnification. Scale bar is 10 μm. (**e**) Image of dentin region at 5 KX magnification. Scale bar is 1 μm. (**f**) Image of dentin region at 10 KX magnification. Scale bar is 1 μm.

To better understand the effectiveness of the SiS ZnO treatment of the tooth sample, we broke the tip of the tooth as illustrated in Fig. 3a. We chose to image the broken tip along the face that should have had no direct exposure to the ZnO precursor gases. The only way there should be any ZnO or conductivity is if the precursors were able to penetrate through the dentin tubules connecting to the pulp region, or other nanoporous channels in the dentin or enamel. Figure 3c–f, shows a sequence of SEM micrographs taken at increasing magnification in the dentin region that had no direct exposure to the ZnO precursor gases. We observed that the dentin tubules are about 800 nm to 1 μm in diameter, and there is no evidence of charging in the acquisition of the image.

### Electron imaging of a SiS ZnO treated brain tissue sample

**Figure 4:**
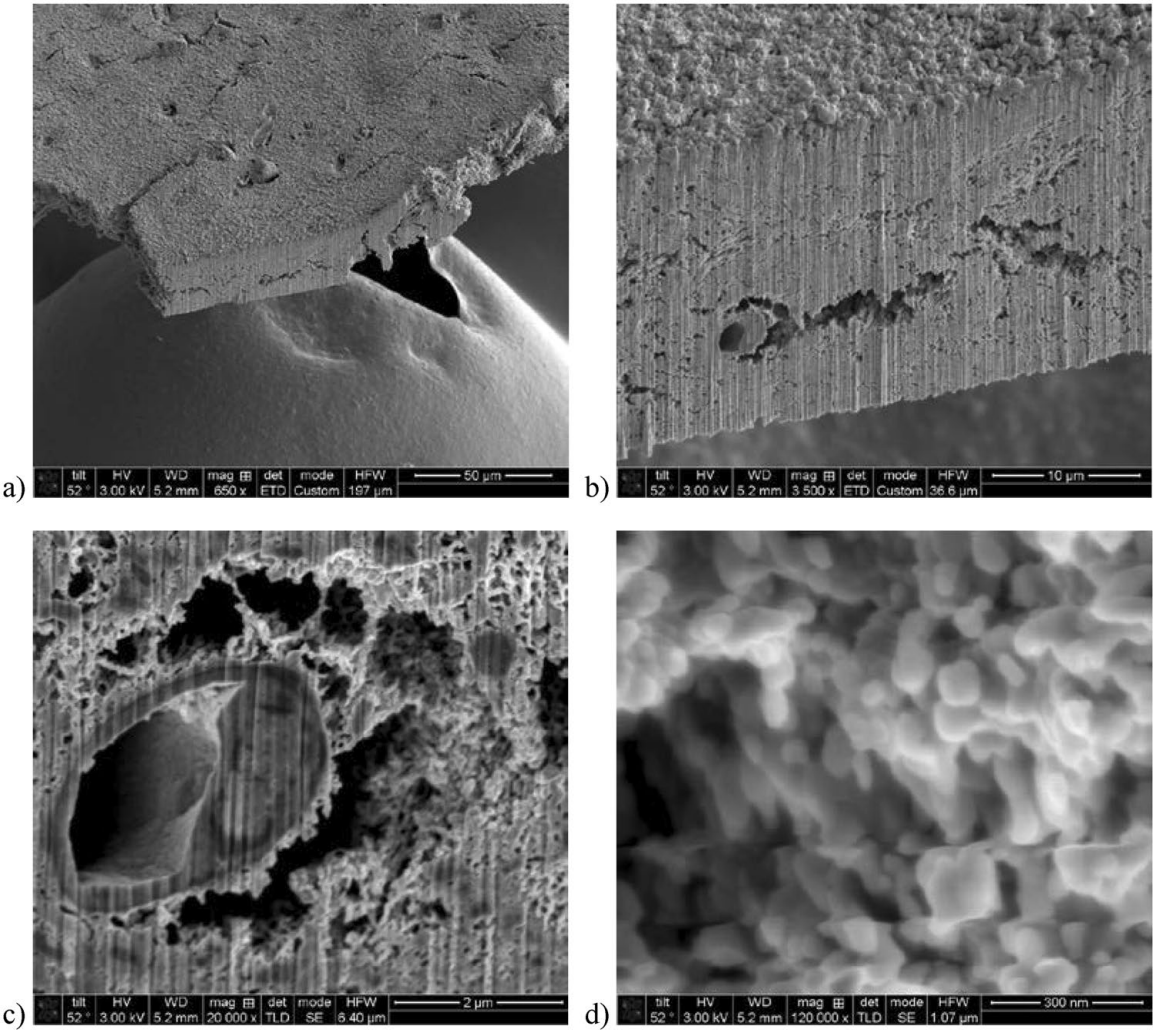
SEM micrographs of brain tissue sample after SiS ZnO treatment and after a slice was milled off using focused ion beam. Vertical striations are artifacts from the ion beam milling procedure. (**a**) SEM micrograph image at 650 X. Scale bar is 50 μm. (**b**) SEM micrograph image at 3.5 K X. Scale bar is 10 μm. (**c**) SEM micrograph image of a sliced putative cell (neuron) at 20 KX. Scale bar is 2 μm. (**d**) Image taken at one of the smaller cavities above the putative cell (neuron) seen in (**c**) at 120 KX. Scale bar is 300 nm.

In order to demonstrate that this technique can be also used for soft biological tissues we tested it on a treated mouse brain cortex tissue sample. The sequence for imaging a mouse brain tissue sample is as follows. The tissue samples were microtomed in 20 to 50 micron thick slices. Then they were fixated with standard biological fixing protocols and super critical CO_2_ drying instead of resin fixation, to maintain sample porosity. The dried samples were then attached to a silicon carrier substrate by double-sided carbon tape and oven pre-treated as the tooth sample described above. After SiS ZnO treatment (18 cycles, which is equivalent to 2–3 nm coating on a flat surface) the sample was imaged in a FEI Dual beam FIB/SEM. To determine infiltration into the tissue, a 100 micron long section was milled off the edge of the sample, revealing a surface that was not directly exposed to the ZnO precursor gases, Fig. 4. We were able to get high resolution images at up to 120 KX magnification (Fig. 4d) and clearly demonstrate nanoscale resolution. Figure 4b,c) highlight a putative cell (neuron) that was sliced in half. The fine structures seen in Fig. 4b–d are small process of other neurons and non-neuronal cells. Considering that we used the coating equivalent of only 2 to 3 nm of ZnO, the structures seen in Fig. 4d cannot be considered aggregations of ZnO nanocrystals, but instead are true neuronal tissue structures. None of the images show charging background found when using the typical epoxy resin fill^17^.

### X-ray imaging of the SiS ZnO treated tooth sample

The tooth sample, in particular, showed significantly improved contrast when imaged by nanoscale X-ray full-field microscopy and nanotomography. The Zn K shell (1s) core electron binding energy is 9.659 keV, is within the optimal illumination region (6–12 keV) for the Hard X-ray Nanoprobe Beamline at the Advanced Photon Source^18, 19^. Imaging of carbon and calcium samples with hard X-rays offers the advantage of large penetration depth into and through samples, but suffer from extremely small absorption contrast^20–23^. Soft X-rays (near the carbon K absorption edge at 284.2 eV), and intermediate X-rays (near the calcium K absorption edge at 4.038 keV) offer good contrast, but suffer from very small sample sizes in order to achieve high resolution images. For example, a zone plate based transmission X-ray microscope (TXM), that can achieve resolutions approaching 20 nm, has a focal depth of the optics that is very small (only 400 nm at 300 eV, and just 5 µm at 4 keV). At 10 keV, the field of view at this beamline is around 15 microns with a 15 micron focal depth, which allows much larger sampling volume than with the loser energy, higher resolution TXMs.

**Figure 5:**
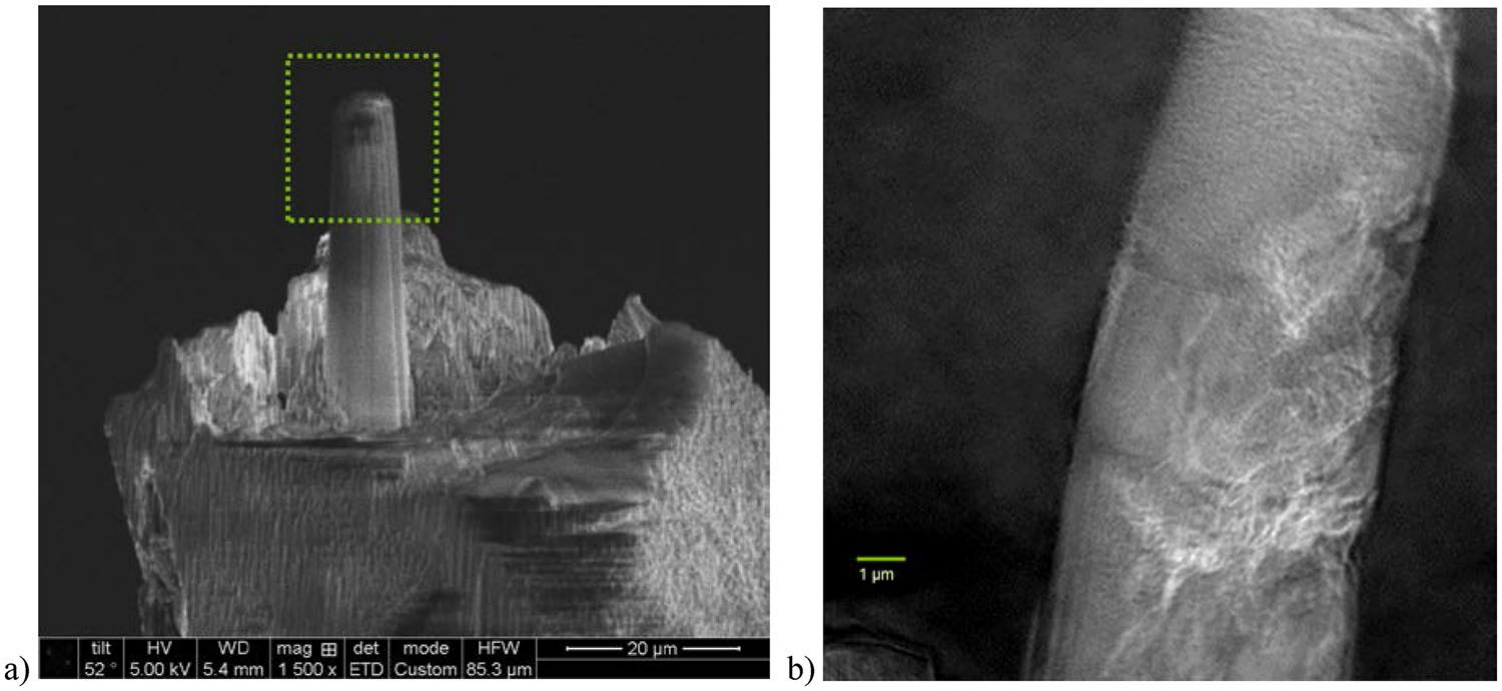
Preparation and X-ray image of tooth sample. (**a**) SEM micrograph of focused ion beam milled piece of tooth sample prepared for high resolution X-ray imaging. Dotted rectangle indicated region imaged. (**b**) First 10 keV X-ray absorption image of another tooth sample using the Zn K edge (1s) for contrast enhancement.

To demonstrate the contrast enhancement of the SiS ZnO treatment we removed a small piece near the root of the tooth and focus ion beam milled cylindrical sections for imaging, Fig. 5a, that were about 8 micron in diameter and over 20 micron tall. The full-field projection image in Fig. 5b clearly shows detailed internal cracks and morphology not observable with electron microscopy.

**Figure 6:**
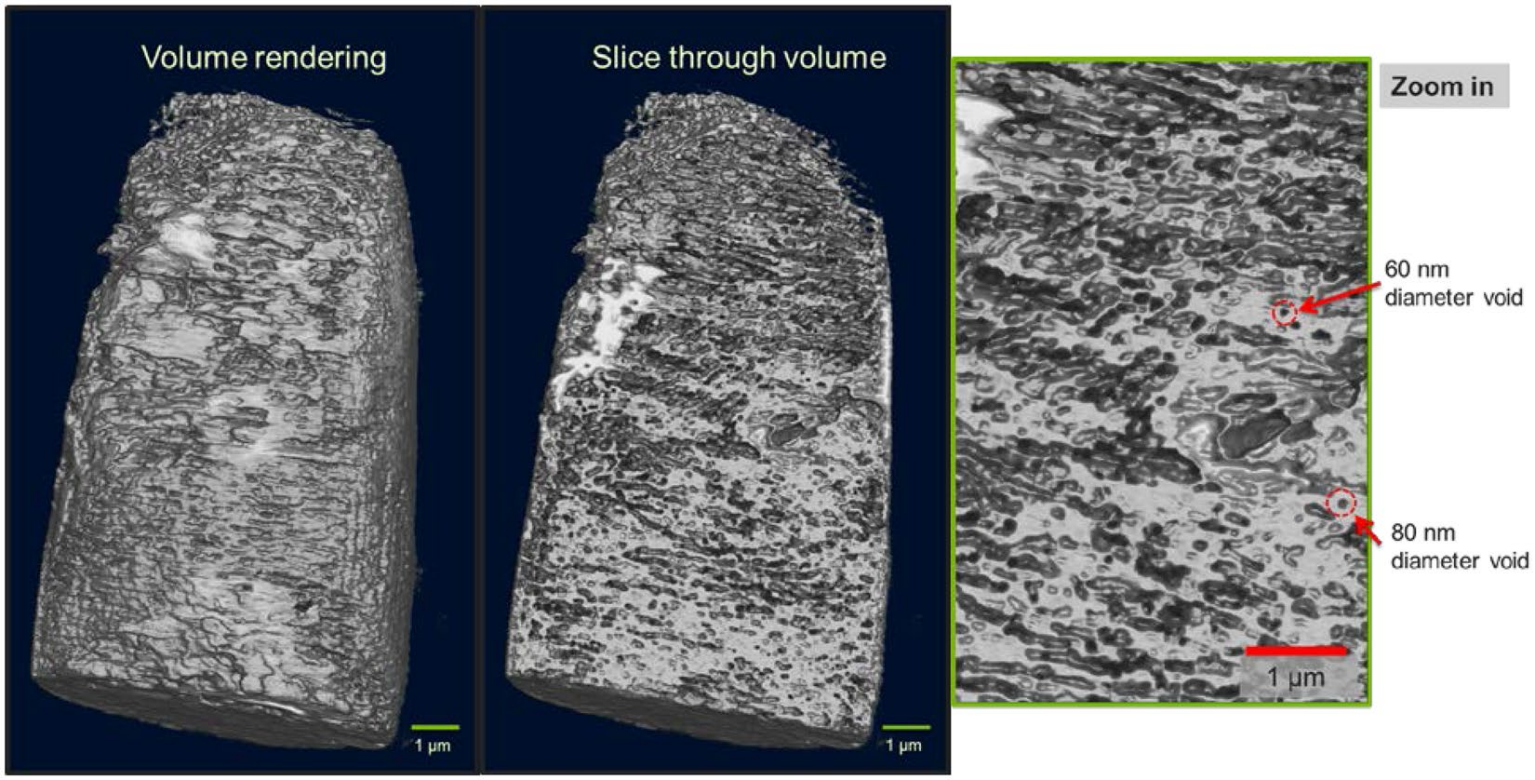
X-ray tomography imaging of tooth sample using SiS ZnO as contrast enhancement. Reconstruction results showing sequence of total volume rendering, a slice through the volume, and a zoom into the section of the slice highlighting the presence of detailed nanoscale features and well-defined pores as small as 60 nm to 80 nm in diameter within the tooth.

In addition, we performed X-ray nanotomography of a tooth sample using the SiS ZnO to enhance the contrast of the interior structure. A full set of projection images (1.5 second exposures, 0.5 degree steps, with 10 images averaged per projection to increase feature contrast) was collected and the data was reconstructed using and algebraic reconstruction technique^24^ to obtain a volume reconstruction of the tooth sample, Fig. 6. The 3D volume rendering confirms the electron microscopy views of the exterior of the sample, and with the nanotomography data we are able to view the *interior* morphology of the tooth sample, Fig. 6b,c. The reconstruction clearly defines small features and pores as small as 60 nm to 80 nm across inside of the tooth, noticeably enhanced by the ZnO SiS treatment. Given the large absorption cross section of Zn, and the ability of the ZnO precursor gases to penetrate deeply into the tooth sample, we have shown that SiS ZnO treatments can be valuable enhancement methods for X-ray imaging of biological samples.

## Discussion

Although we have shown the ability to obtain high resolution electron microscopy images using SiS ZnO, it is important to keep in mind that ZnO is not a metal but a semiconductor. As such the conductivity is not as high as that of a metal such as gold. Therefore images have to be taken in a slightly different manner than when using a metal coating. ZnO is conductive *enough* to dissipate charge. For images with magnifications greater than 20 KX it is best to use fast integration scans. This reduces the amount of charging that develops on the sample. Also, it is important to provide a good grounding of the sample to the electron microscope. This dictates the use of a quality conductive tape path from the silicon sample to the sample holder to help with charge dissipation.

In terms of future applications for enhanced x-ray imaging, there is a need for imaging at X-ray energies of 30 keV and above for large sample 3D imaging^25, 26^. Although ZnO has been demonstrated in this paper to be an excellent contrast enhancement agent for 10 keV x-rays, tin oxide (SnO) could be the candidate for 30 keV x-rays and above. SnO can be synthesized in an ALD tool at temperatures as low as 50 °C^15^ and the K (1 s) shell of Sn has a value of 29.2 keV.

In summary, we have shown that ZnO metal oxide infiltration can be useful for high resolution imaging of biological samples in both electron and X-ray microscopy. The method is compatible with standard fixation techniques that leave the sample dry, such as finishing with a super critical CO_2_ drying. We have demonstrated this technique on tooth and brain tissue samples. We also have shown high resolution X-ray nanotomography that can utilize the enhanced contrast available above the Zn K (1s) absorption edge, obtaining the first 10 keV nanoscale X-ray absorption images of tooth samples.

We believe there are major opportunities for this technique beyond biological samples such as shale rock, sandstone, concrete^27^, and others. For example, understanding the role of porosity and permeability is critical for understanding the flow of fluids in rock bodies involved in fracking and gas storage (e.g. CO_2_ sequestration). The combination of ZnO infiltration and X-ray imaging can provide non-destructive enhanced imaging capabilities to the biomedical, dental, construction, and oil communities.

## Methods

The work performed did not involve live vertebrates and involved the handling of a canine tooth and fixed mouse brain tissue. Upon review, the work was graded as biosafety level 1 by Argonne’s Institutional Biosafety Committee and all procedures were followed in accordance with our institutional guidelines. No human derived material, samples known to be infectious, or organism containing recombinant DNA was used.

All animal experiments were conducted in accordance with University of Chicago and internationally-accepted standards. Prior approval for all experiments was obtained from the University of Chicago IACUC committee (permit no: 7248; iacuc@uchicago.edu).

For the SiS treatment the samples were pre-treated in a vacuum oven, initially at room temperature, ramped to 95 °C, and then baked at 95 °C for 4 to 8 hours. Both tooth and brain tissue samples were inspected by optical microscopy before and after the vacuum oven pre-treatment to determine if the samples were compatible with the SiS process. Given that no sample changes were observable, we proceeded with running a SiS ZnO process. No further processing was needed for both electron and X-ray microscopy imaging.

The temperature of the vacuum pre-treatment matches that of the SiS ZnO process used in our Arradiance Gemstar-8 ALD tool. The Arradiance tool allows for exhaust valve shutoff during precursor exposure to the sample, which is critical for the infiltration process to proceed, as it allows the precursors time to diffuse into the polymer matrix. The SiS process is carried out at 95 °C. The precursors used were DI water (H_2_O) and diethylzinc (DEZ), which was purchased from Strem Chemical. We used 18 cycles of [H_2_O:DEZ] for ZnO SiS processing of the samples of 18 cycles of [H_2_O:DEZ], which would be the equivalent of a 2–3 nm coating on a flat surface. A half cycle, [H_2_O] or [DEZ], consists of precursor injection in a series of short pulses of the precursor, followed by 2 to 4 minutes of precursor residency in the chamber, and a nitrogen flush for 40s followed by a 3s pump chamber down prior to the next injection. In this case eleven short pulses of H_2_O and fourteen pulses of DEZ were injected per half cycle. The residency time for H_2_O was 2 minutes and that of DEZ was 4 minutes. The difference is due to the different diffusion rates of the precursor gases.

### Data availability statement

Most of the data generated or analyzed during this study are included in this published article (and its Supplementary Information file). The rest of the datasets generated during and/or analyzed during the current study are available from the corresponding author on reasonable request.

## Electronic supplementary material

Supplementary Information
