# Supplementary material for: Contrast enhancement of biological nanoporous materials with zinc oxide infiltration for electron and X-ray nanoscale microscopy
Source: Sci Rep. 2017 Jul 19;7:5879. doi: 10.1038/s41598-017-05690-6 (PMC5517423; doi:10.1038/s41598-017-05690-6)
Supplement: Supplementary file 1 — Supplementary Information [file 41598_2017_5690_MOESM1_ESM.pdf]

Supplementary Information

Contrast enhancement of biological nanoporous materials with zinc oxide infiltration for electron and X-ray nanoscale microscopy

L. E. Ocola [+, 1,*], V. Sampathkumar [2], N. Kasthuri [2, 3], R. P. Winarski [+, 1]

[1]Argonne National Laboratory, Center for Nanoscale Materials, Argonne, 60543, USA
[2]University of Chicago, Department of Neurobiology, Chicago, 60637, USA
[3]Argonne National Laboratory, Nanoscience and Technology Division, Argonne, 60543, USA
[*] ocola@anl.gov
[+] These authors contributed equally to this work

Diffusion control experiments

An initial test to determine the effectiveness of SiS ZnO diffusion in a porous media consisted of measuring the penetration depth into 1 mm thick samples of PDMS (Polydimethylsiloxane). PDMS is commonly used in microfluidic devices and is known to permeate oxygen. Figure S1 summarizes results of this first control experiment. In this experiment H_2O and DEZ were allowed to diffuse for 2 to 4 minutes per cycle. Longer residence times will allow the precursors to diffuse deeper into the material.

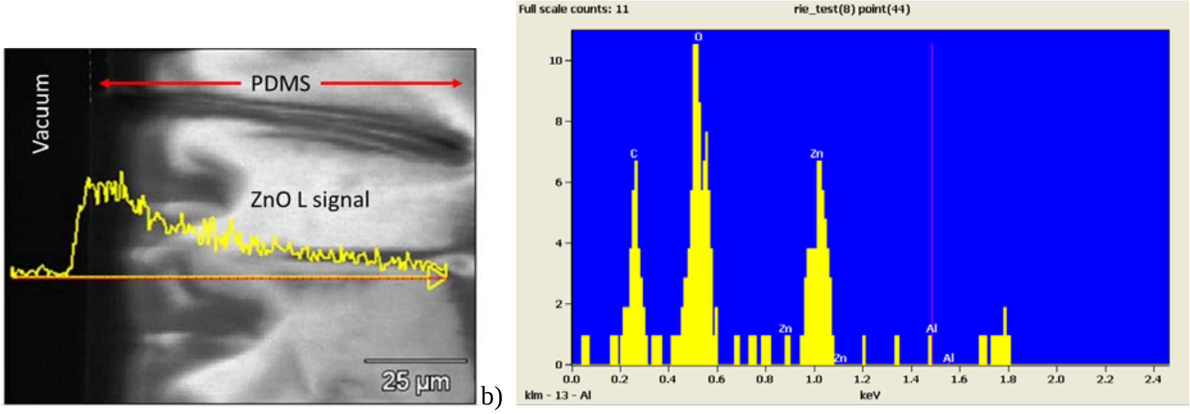

a) b)

c)

Composition at Surface			Composition 25 um deep			Composition 50 um deep		
Element Line	Element Wt.%	Atom %	Element Line	Element Wt.%	Atom %	Element Line	Element Wt.%	Atom %
C K	9.06	17.48	C K	15.36	26.23	C K	25.25	35.07
O K	44.91	65.02	O K	48.78	62.52	O K	57.29	59.73
Al K	2.37	2.04	Al K	0	0	Al K	2.04	1.26
Zn L	43.66	15.47	Zn L	35.86	11.25	Zn L	15.42	3.93
	----------	----------		----------	----------		----------	----------
Total	100	100	Total	100	100	Total	100	100

Figure S1. (a) Cross section image of a SiS treated PDMS sample. Sample was cleaved after treatment. Yellow line is the EDS data from the Zn L-shell. Penetration depth is shown to be greater than 50 microns. (b). EDS spectrum near surface. (c) Normalized atomic composition data from spectra at different depths.

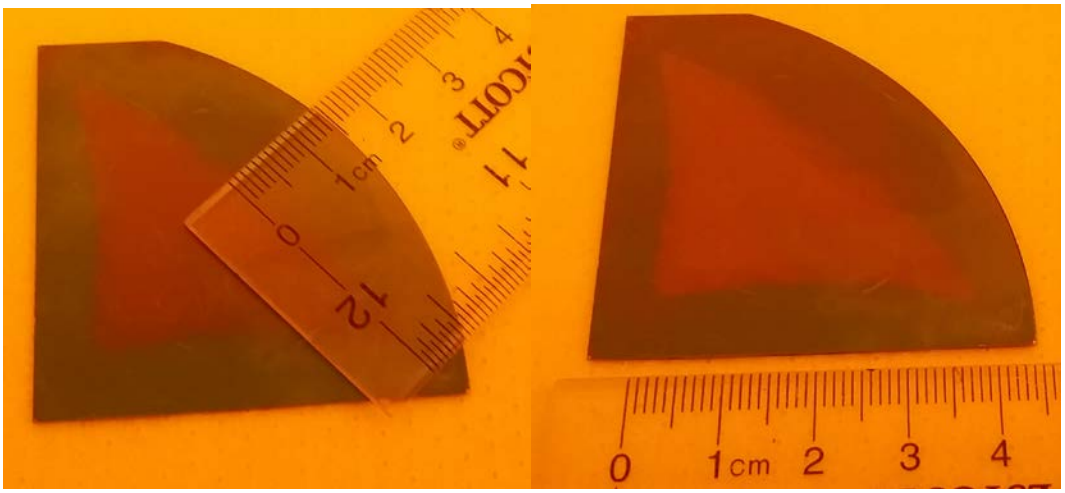

Figure S2. Photos of infiltrated ZnO coating the underside a Si wafer piece. Range is in millimeters. Roughness of the Si wafer back side is of the order of several microns. Similar roughness can be found on the sample holder in the ALD tool. Photo indicates possible infiltration range in highly porous media can reach several millimeters.

Tooth control experiments

Figure S3 shows SEM images of untreated tooth sample. The untreated tooth sample went through the same oven pretreatment at 95 ^{o}C in vacuum as the treated samples. Figure S4 shows EDS spectra from an untreated tooth sample and the same sample after treatment. The plots clearly show that prior to treatment there is no detectable Zn. Figure S5 shows EDS data of sample shown in Figure 3 of manuscript. Note that the Zn –K-shell signal drops to zero once the line scan passes over the edge of the tooth sample. This clearly implies that the noisy data from the tooth is from the infiltrated ZnO and not from any other background sources.

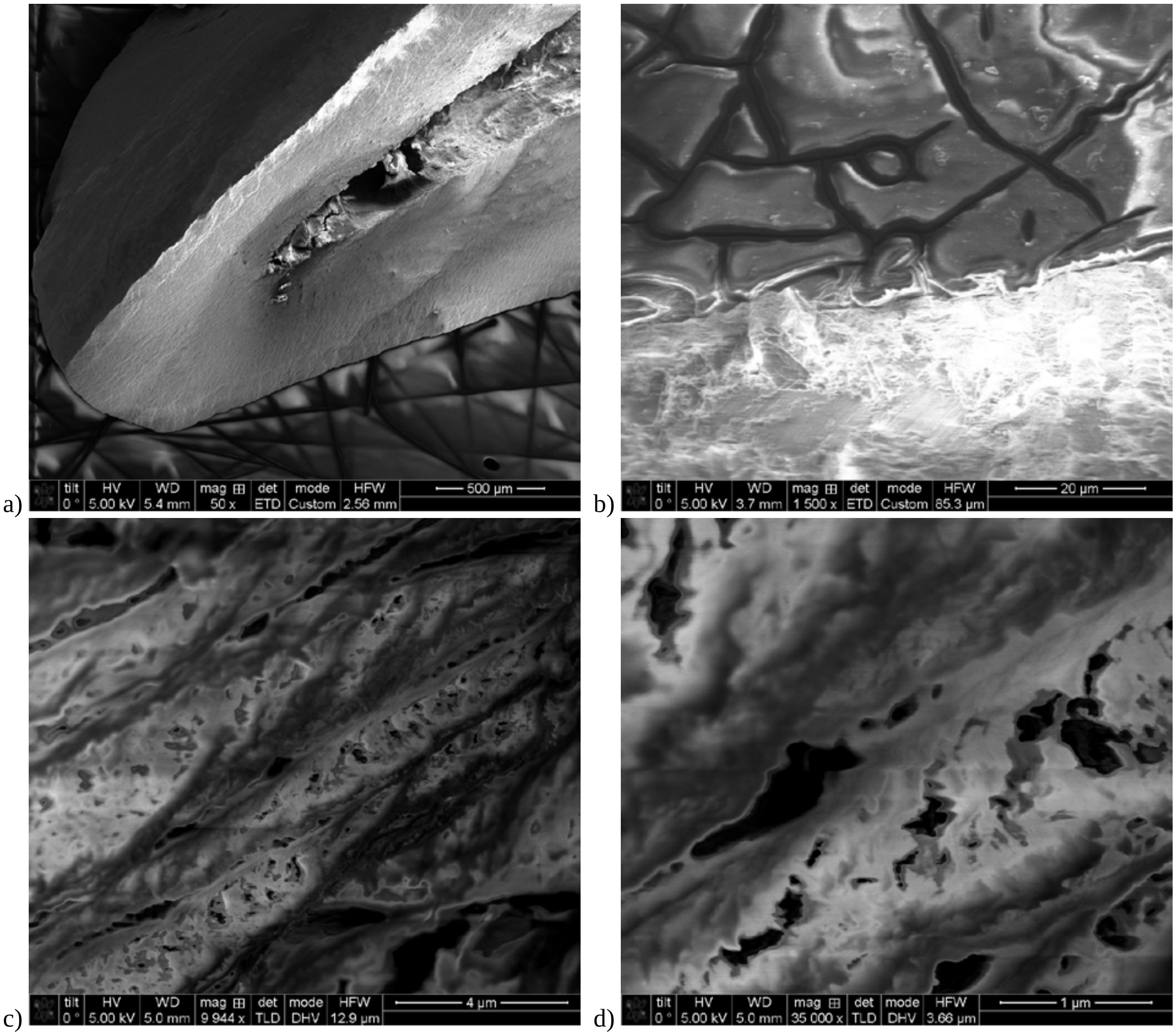

Figure S3. SEM imaging of untreated tooth sample shows clearly effect of charging. a) SEM image at 50 X magnification. b) SEM image of enamel at 1.5 KX magnification shows surface damage and significant change in contrast. c) and d) are higher magnification images that clearly indicate effects of charging.

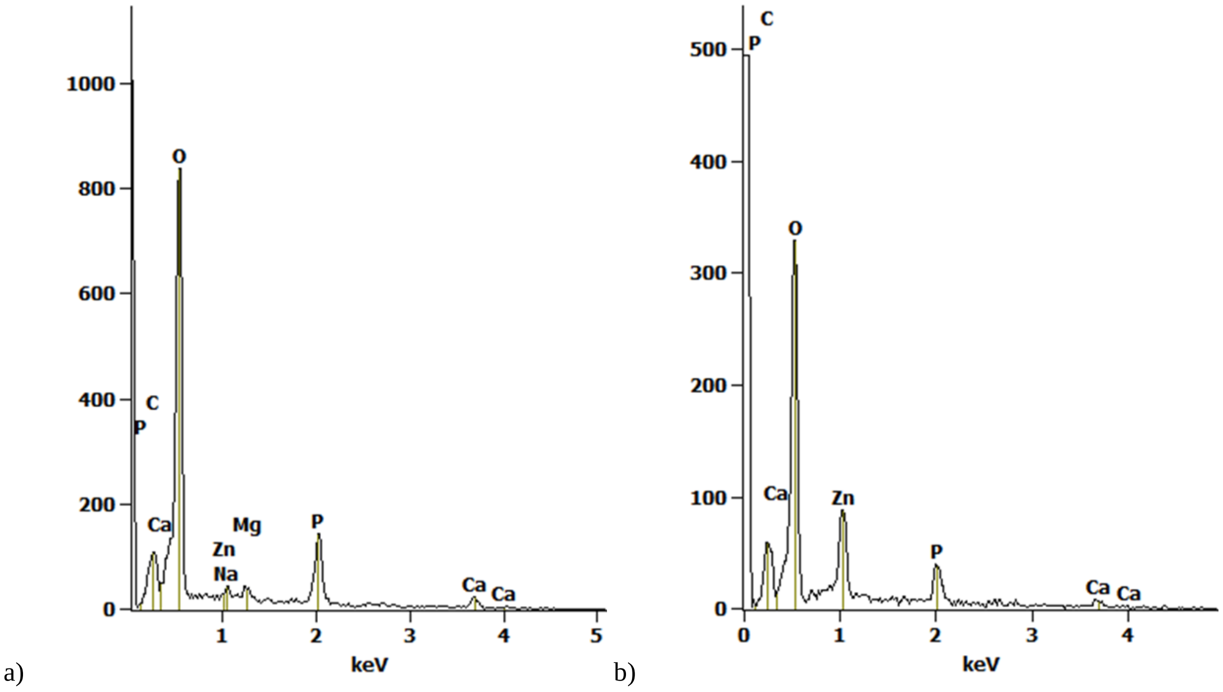

Figure S4. EDS spectra at 5 KeV on the surface of a tooth sample before and after SiS ZnO treatment. a) before treatment shows barely detectable presence of Zn. b) after SiS ZnO treatment shows strong Zn peak.

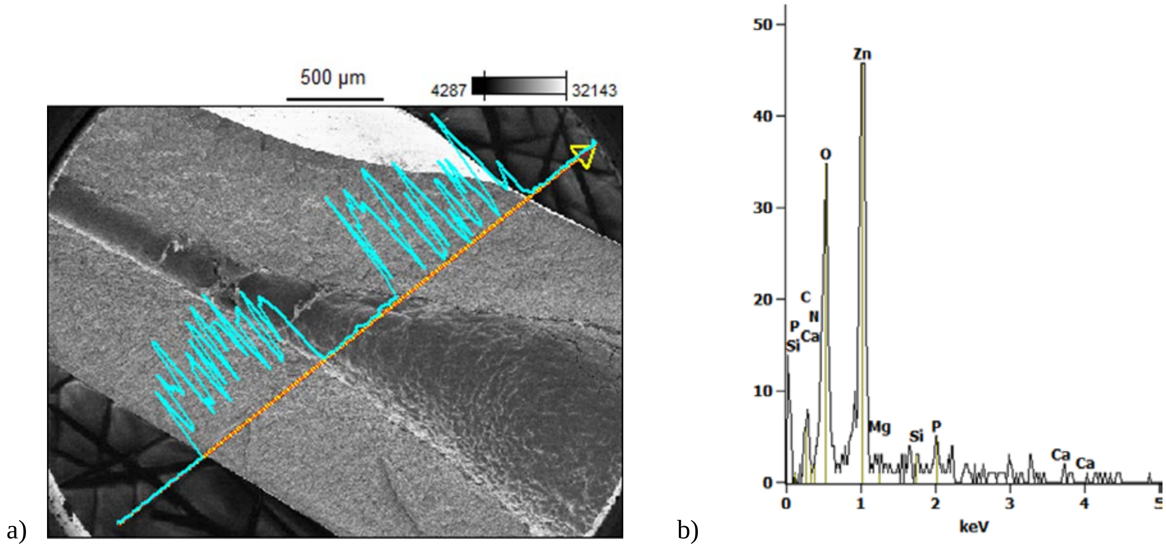

Figure S5. EDS line scan data at 5 KeV of tooth sample after SiS treatment. Cyan line is Zn L-shell data. a) EDS spectra taken across a line over of one of the faces of the tooth sample. b) Spectra at one point on the cyan line. Note Zn L-shell data goes to zero once line is off the tooth.

Neuron sample control experiments

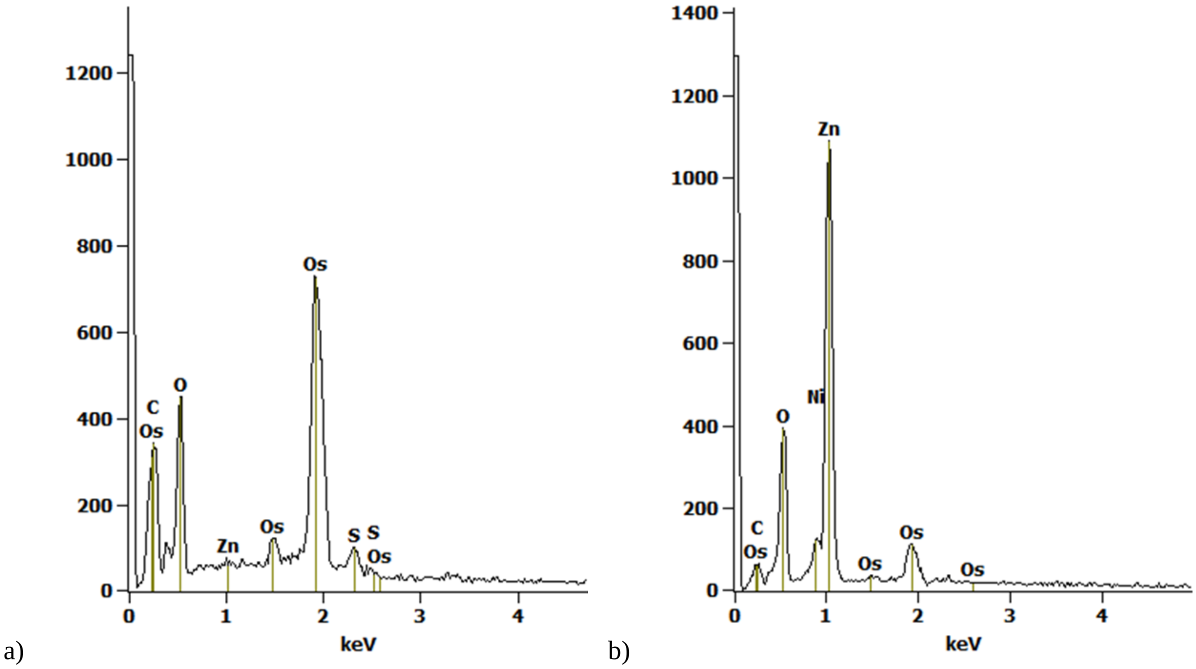

Figure S6. EDS spectra at 10 KeV on the surface of a brain tissue sample before and after SiS ZnO treatment. a) before treatment shows barely detectable presence of Zn. b) after SiS ZnO treatment shows strong Zn peak.

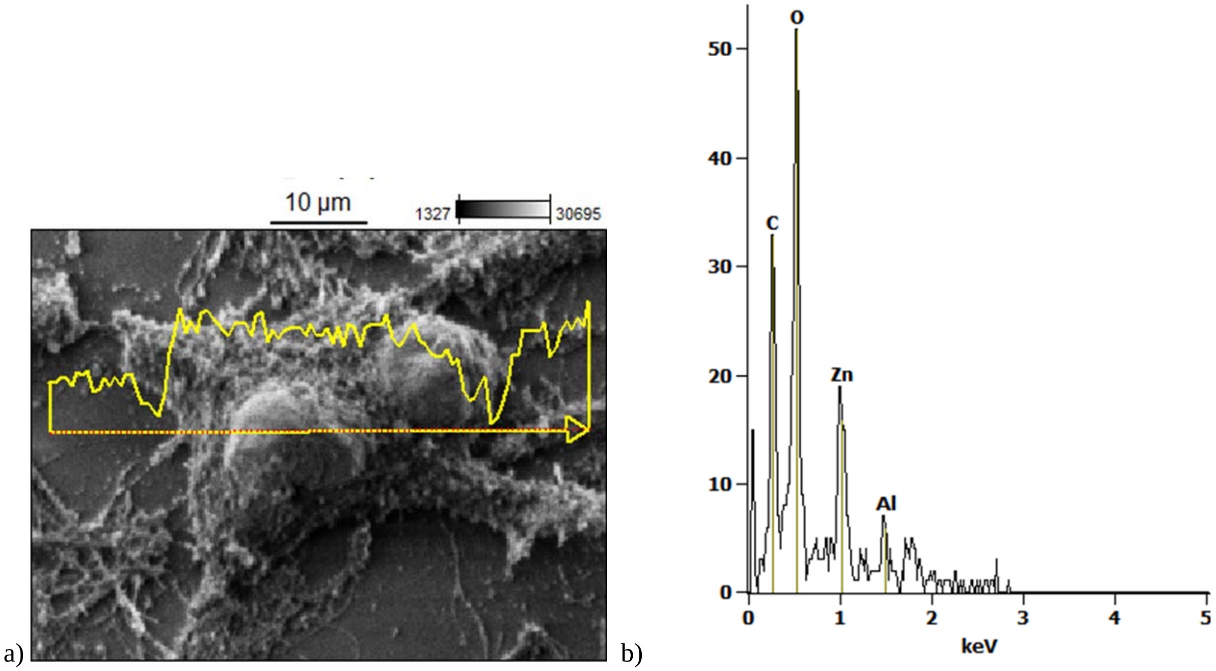

Figure S7. EDS line scan data at 3 KeV of neuron culture on glass sample after SiS treatment. Yellow line is Zn L-shell data. a) EDS spectra taken across a line over a neuron. b) Spectra at one point on the yellow line directly over the neuron.

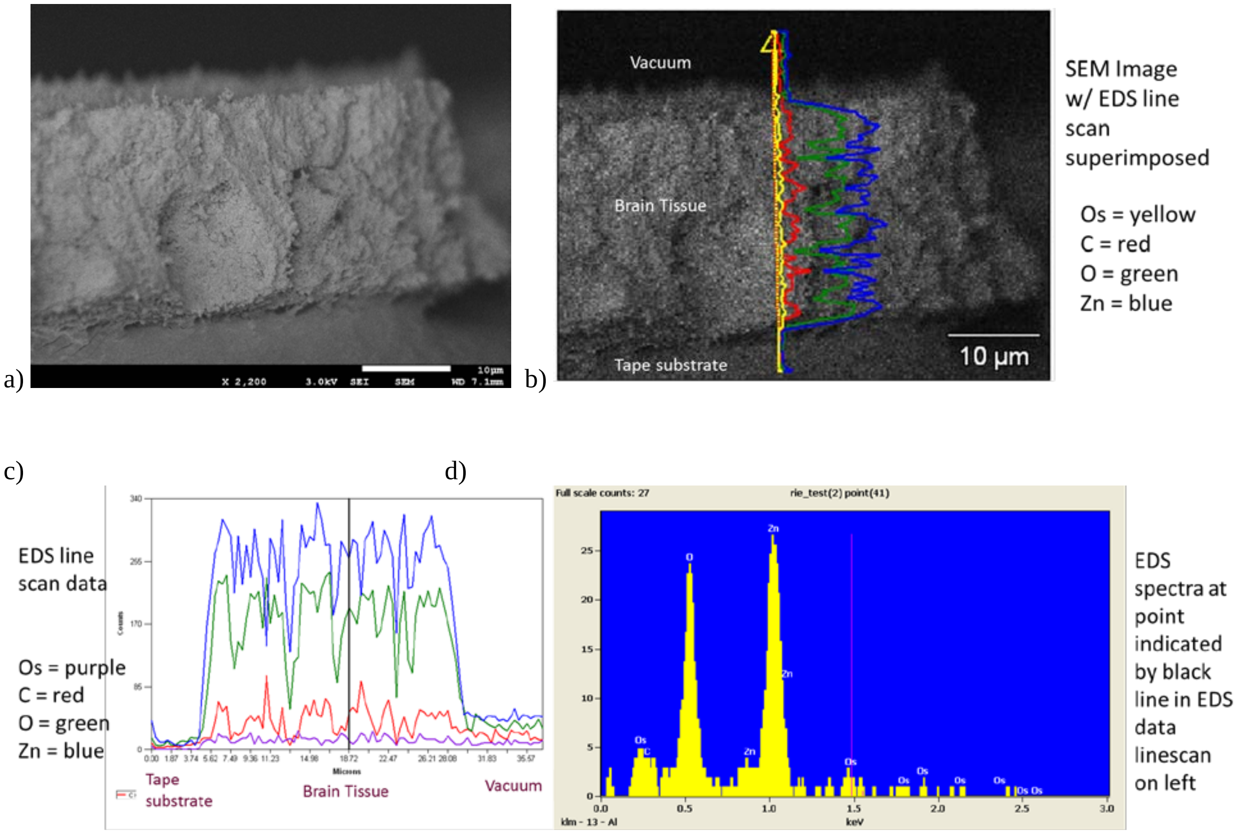

Figure S8. Cross sectional EDS data of brain tissue using JEOL 7500 SEM. a) SEM image of broken piece of brain tissue. b) EDS line scan superimposed to SEM image. c) EDS line scan intensity as a function of position. d) EDS spectra at one point in the brain tissue showing strong Zn K-shell peak at 1 KV.

Image comparison of untreated vs SiS ZnO treated 50 micron thick brain tissue

While osmium staining provides decent charge dissipation that allows SEM imaging at moderate magnifications, evidence of charging is still apparent and treated samples exhibit better imaging quality. Figures S9, S10, and S11 illustrate the similarities and advantage of SiS ZnO treatment for high resolution imaging.

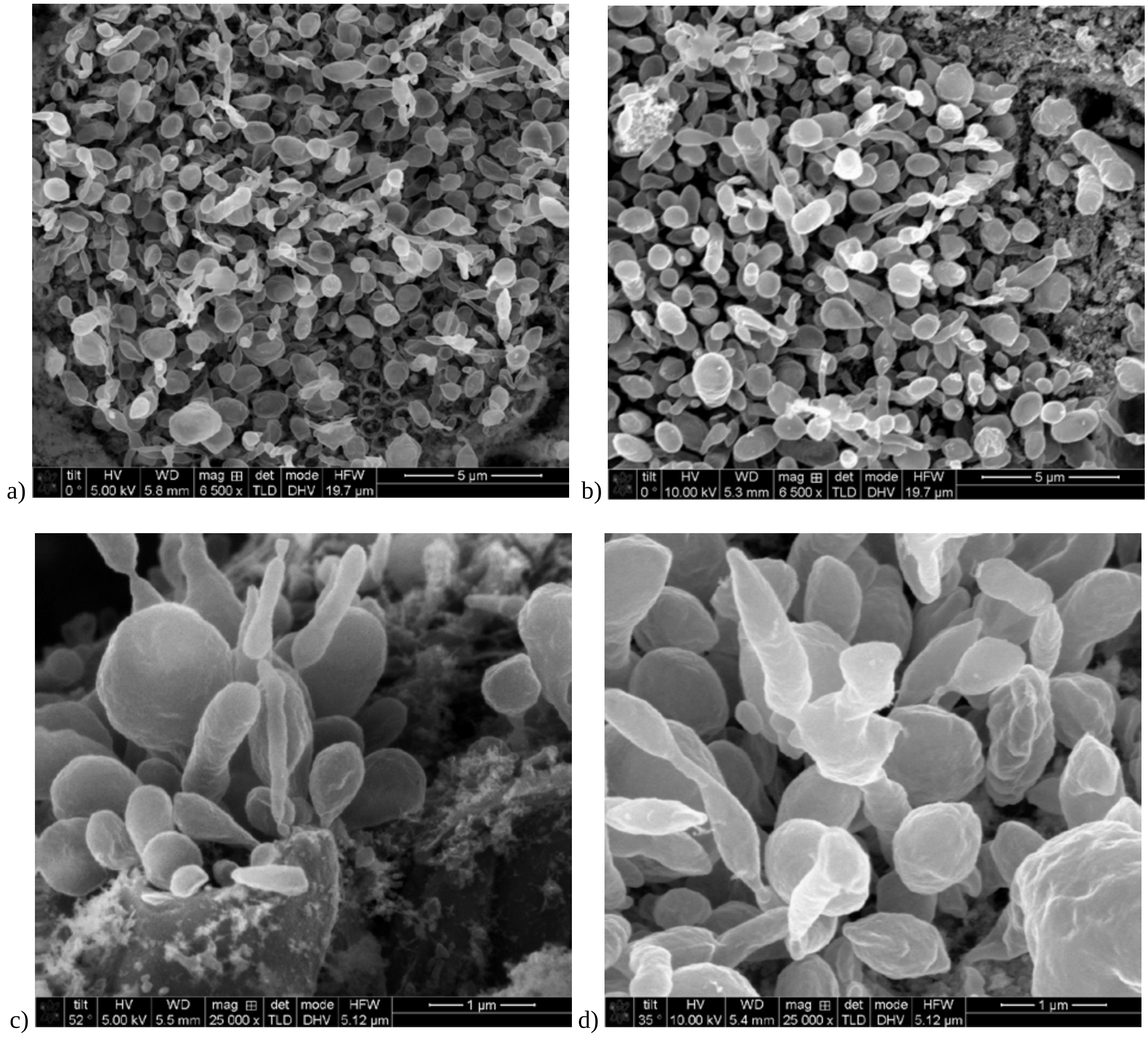

Figure S9. SEM imaging comparison of untreated and SiS ZnO treated brain tissue samples. a) and c) are untreated. b) and d) are treated. a) and b) are imaged at 6.5 KX magnification. c) and d) are imaged at 25 KX magnification. Results are similar, also no evidence of ZnO grains or coating.

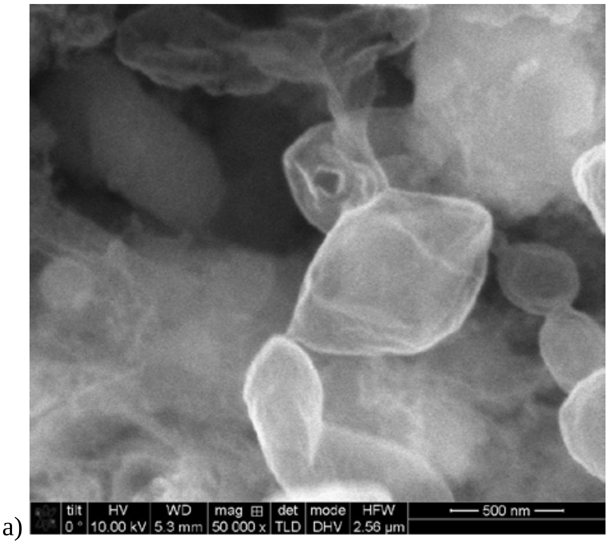

a)

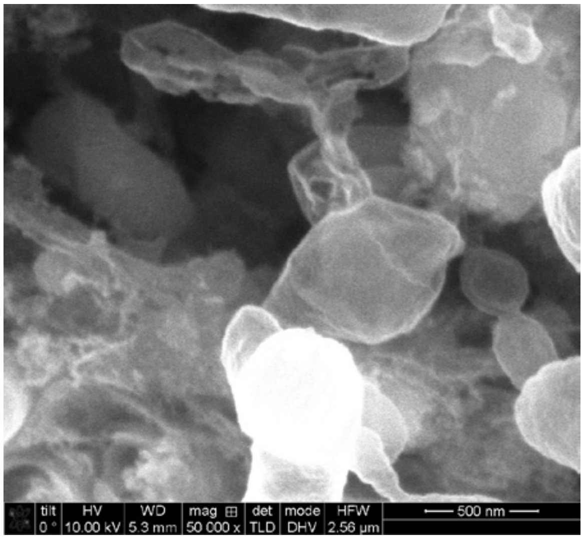

b)

Figure S10. Untreated brain tissue imaged at 50 KX magnification, same location, at different times. a) Snap image at time = 0 s. b) Snap image taken after letting the SEM scan over the region for 10 s. Notice that structures have moved and a new structure appears in the frame at the bottom. It was pulled in from just outside the field of view, i.e. a movement of at least 500 nm.

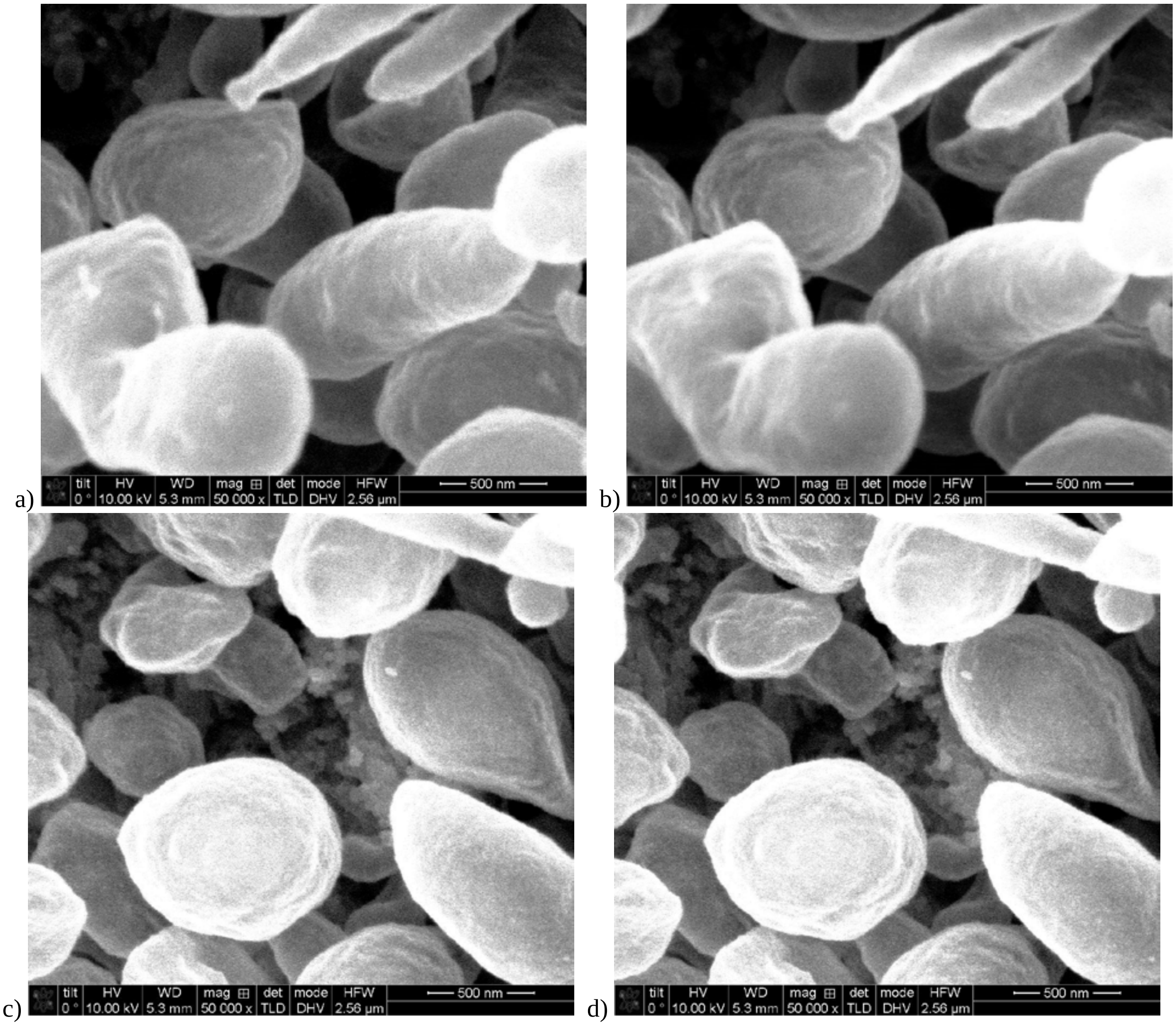

Figure S11. SiS ZnO treated brain tissue imaged at 50 KX magnification, same location, at different times. a) and c) Snap images at time = 0 s. b) and d) Snap images taken after letting the SEM scan over the region for 10 s. Notice that most structures have not moved and one or two barely moved, < 50 nm.
